# Supplementary material for: Characterization of the Heavy-Metal-Associated Isoprenylated Plant Protein (HIPP) Gene Family from Triticeae Species
Source: Int J Mol Sci. 2020 Aug 27;21(17):6191. doi: 10.3390/ijms21176191 (PMC7504674; doi:10.3390/ijms21176191)
Supplement: Supplementary file 1 [file ijms-21-06191-s001.zip › ijms-883890 suppl final/Supplementary figures.docx]

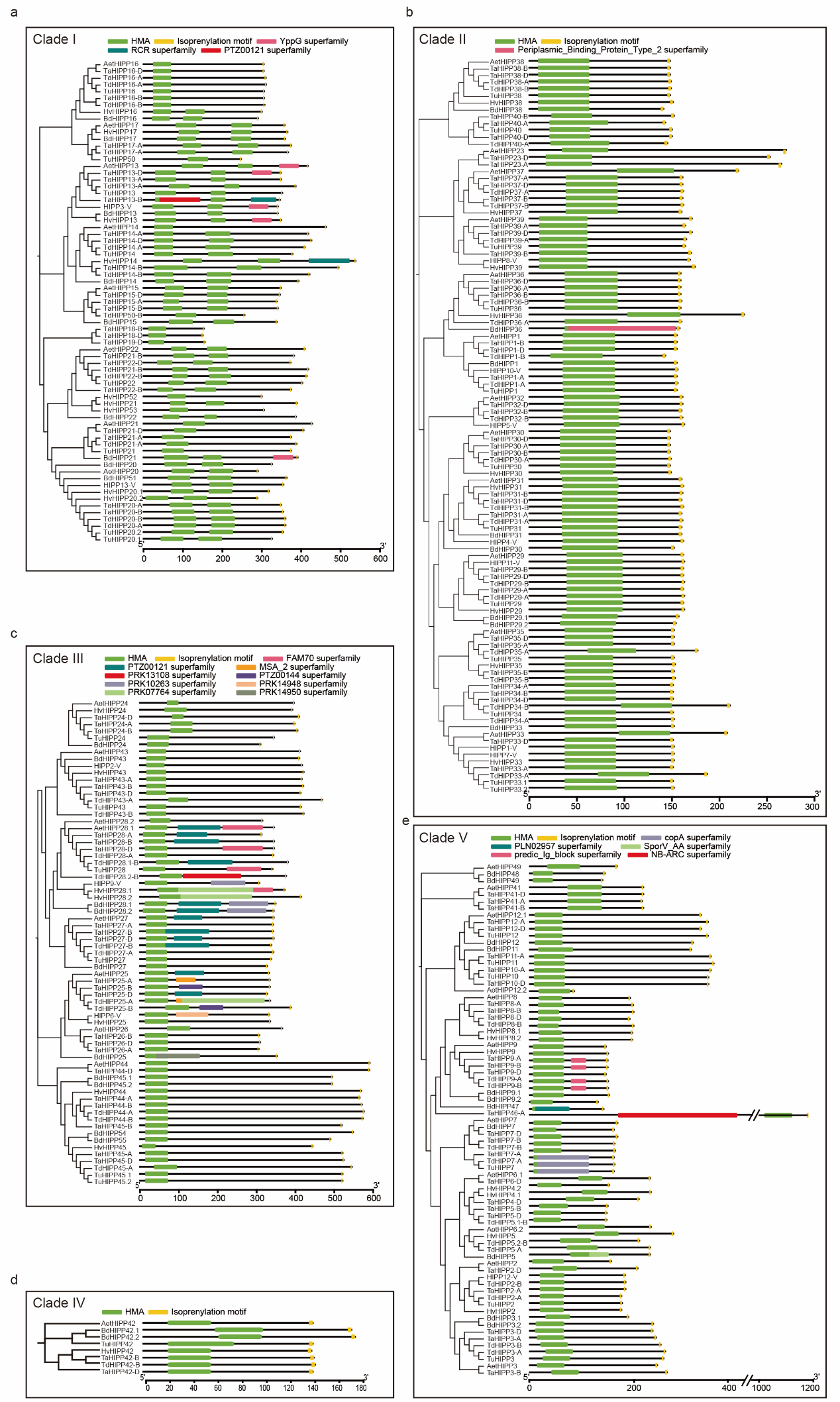


**Figure S1.** Phylogenetic analysis and protein structures of the heavy-metal-associated isoprenylated plant protein (*HIPP*) gene family in common wheat and related *Triticeae* species. a-e indicates clade I- clade V, respectively. The left and the right represent the phylogenetic tree and protein structure, respectively. Multiple protein domains were represented by colored boxes. Legends are shown


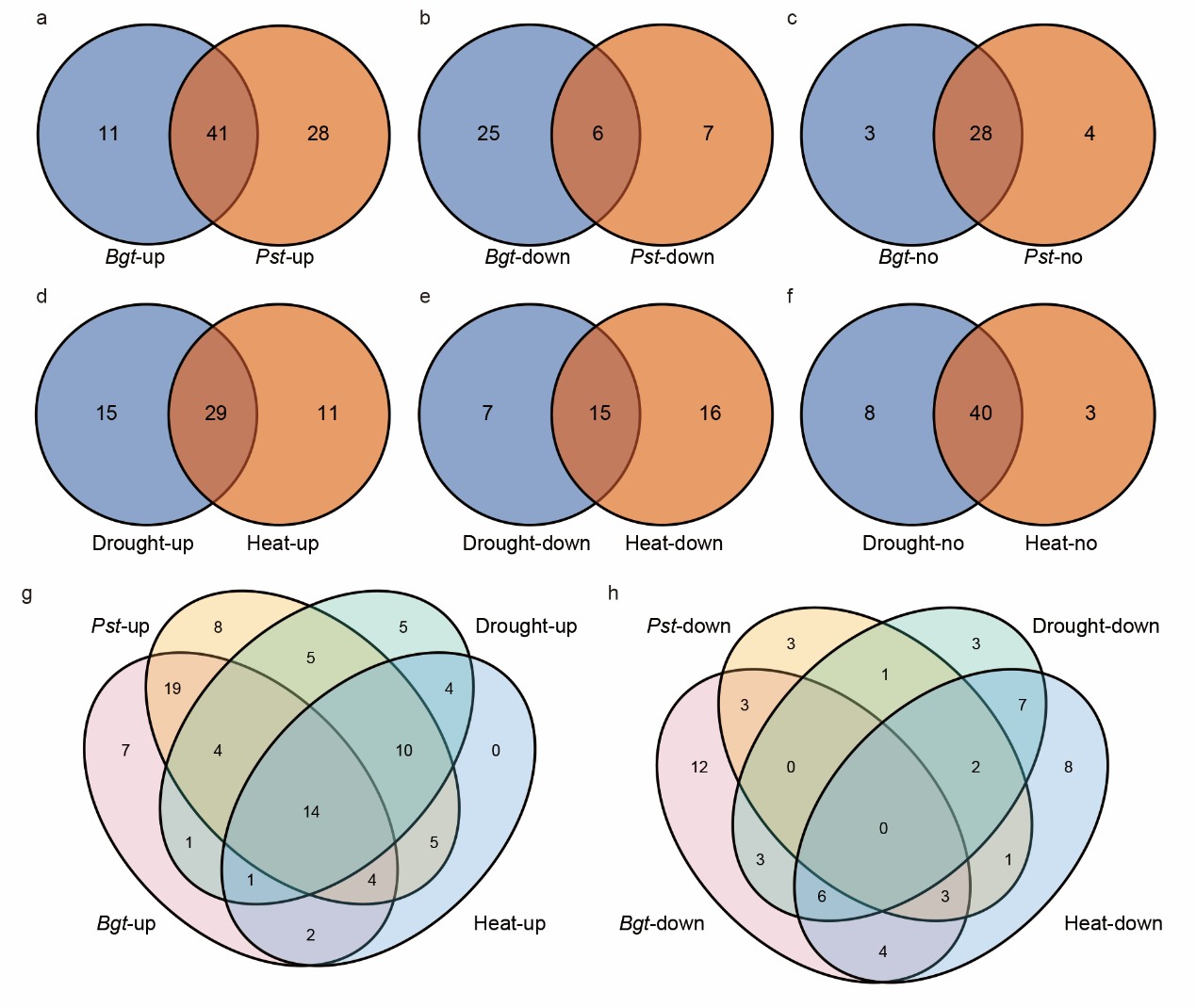


**Figure S2**. Venn diagrams of differentially expressed *HIPPs* under different stresses. (a) *HIPPs* were up regulated under the stress of powdery mildew and yellow rust; (b) *HIPPs* were down regulated under the stress of powdery mildew and yellow rust; (c) *HIPPs* were no obvious expression change under the stress of powdery mildew and yellow rust; (d) *HIPPs* were up regulated under drought and heat stress; (e) *HIPPs* were down regulated under drought and heat stress; (f) *HIPPs* were no obvious expression change under drought and heat stress; (g) *HIPPs* were up regulated under four stresses; (h) *HIPPs* were down regulated under four stresses. Abbreviations: *Bgt*, powdery mildew; *Pst*, yellow rust; Up, up regulated; Down, down regulated; No, no obvious expression change.


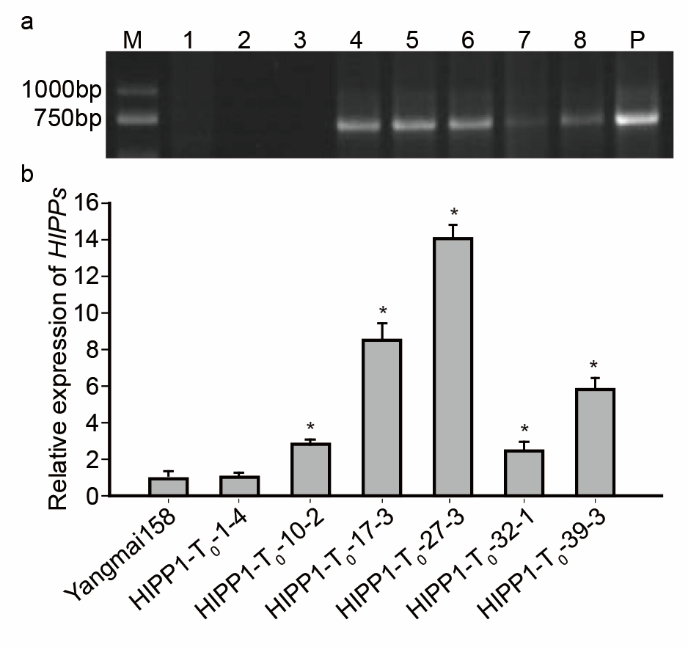


**Figure S3.** Identification of positive transgenic plants for *HIPP1-V.* (a): Specific amplicon for *HIPP1-V* was present in five T_0_ transgenic lines. Yangmai158 and HIPP1-T_0_-1-4 were negative controls, and plasmid *pBI220*-*HIPP1-V* was used as positive control. M: DL2000 DNA ladder; 1: Yangmai158; 2: ddH_2_O; 3: HIPP1-T_0_-1-4; 4: OE-HIPP1-T_0_-10-2; 5: OE-HIPP1-T_0_-17-3; 6: OE-HIPP1-T_0_-27-3; 7: OE-HIPP1-T_0_-32-1; 8: OE-HIPP1-T_0_-39-3; P: pBI220-HIPP1-V plasmid. (b): *HIPP1-V* expression in five *HIPP1-V* T_0_ positive transgenic lines (OE-HIPP1-T_0_-10-2, OE-HIPP1-T_0_-17-3, OE-HIPP1-T_0_-27-3, OE-HIPP1-T_0_-32-1 and OE-HIPP1-T_0_-39-3) and negative controls (HIPP1-T_0_-1-4 and Yangmai158).
